# Behavior of PMMA Denture Base Materials Containing Titanium Dioxide Nanoparticles: A Literature Review

**DOI:** 10.1155/2019/6190610

**Published:** 2019-01-17

**Authors:** Mohammed M. Gad, Reem Abualsaud

**Affiliations:** ^1^Lecturer, Department of Substitutive Dental Sciences, College of Dentistry, Imam Abdulrahman Bin Faisal University, P.O. Box 1982, Dammam 31411, Saudi Arabia; ^2^Assistant Professor, Department of Substitutive Dental Sciences, College of Dentistry, Imam Abdulrahman Bin Faisal University, P.O. Box 1982, Dammam 31411, Saudi Arabia

## Abstract

Titanium dioxide nanoparticles (TiO_2_NP) have gained interest in the dental field because of their multiple uses in addition to their antimicrobial effect. One of the applications in dentistry involves the incorporation into poly methyl methacrylate (PMMA) resin. However, there is a lack of evidence on their effects on the behavior of the resulting nanocomposite. Therefore, the present review aims to screen literatures for data related to PMMA/TiO_2_ nanocomposite to figure out the properties of TiO_2_ nanoparticles, methods of addition, interaction with PMMA resin matrix, and finally the addition effects on the properties of introduced nanocomposite and evidence on its clinical performance. Regardless of the latest research progress of PMMA/TiO_2_ nanocomposite, the questionable properties of final nanocomposite and the lack of long-term clinical evidence addressing their performance restrict their wide clinical use. A conclusive connection between nanoparticle size or addition method and nanocomposite properties could not be established.

## 1. Introduction

The numerous advantages of poly methyl methacrylate (PMMA) make it the most dominant polymer used as denture base material. The ease of processing, low cost, light weight, stability in the oral cavity, and aesthetic properties are of these advantages [1]. However, this material is not ideal in every aspect. PMMA resin denture base material has poor surface properties and weak mechanical properties including impact and flexural strengths [2]. Therefore, resins should be reinforced using different materials to enhance their properties. Recently, nanotechnology invaded the dental field and initiated investigative research projects to explore the possible applications and expected benefits within dentistry. It is of paramount importance to know the science behind this nanotechnology to know how to utilize it to our advantage. Polymeric nanocomposites are made of polymer matrix and filler at the nanoscale [3]. Literature shows that nanoscale reinforcing agents produce new mechanical and physical properties, which create a new class of nanocomposites [4]. In dentistry, many attempts have been made to create an improved version of PMMA with the addition of different nanosized fillers [5]. The properties of the new composite material depend on the nature of the added nanoparticles, their size, and morphology [6].

The science and applications of nanotechnology are constantly evolving as we witness new products being introduced into the market. This comes with great responsibility to insure the safety, efficiency, and applicability of such new technologies. Although nanomaterials generally offer superior properties, their mechanical properties fall short in comparison to pure materials. Lately, worldwide research showed several advancements in the nanocomposite field after the extensive research on mechanical and physical properties of these nanocomposites [7]. Different nanoparticles have been incorporated into the polymer matrix. Among these is TiO_2_NP with its unique properties [7, 8]. The literature investigated the effect of TiO_2_NP on some properties of PMMA. But a comprehensive study on the overall performance of PMMA nanocomposite was not observed. In addition to that, the relation between final properties of the composite and the actual structure is not available. One of the goals of the present study is to review the effectiveness of TiO_2_NP addition to PMMA for dental applications. It also seems necessary to determine the best percentage of filler required to improve the properties as well as the method of reinforcement to control future applications.

**Figure 1:**
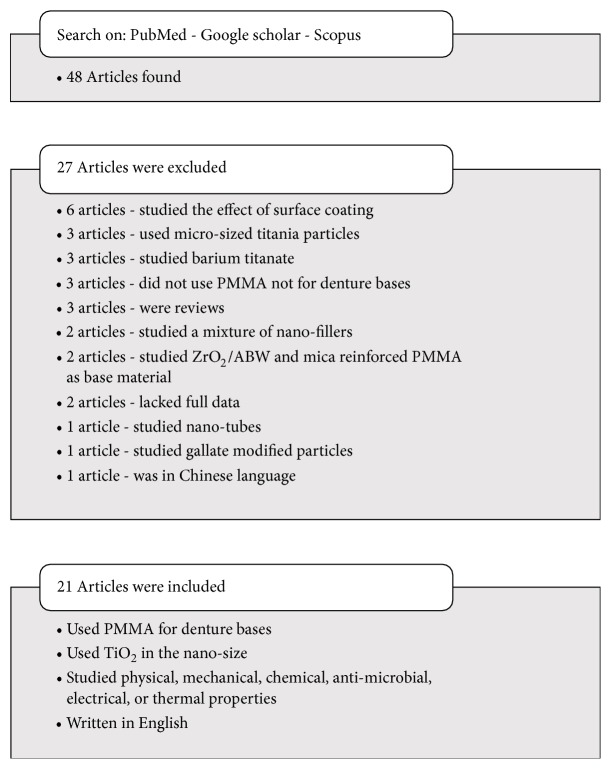
Study design.

Up to November of 2018, database search on Google scholar, PubMed, and Scopus was conducted using the key words “Denture base; Polymethylmethacrylate (PMMA); TiO_2_ nanoparticles; Physical properties”. Forty-eight articles were found (Figure 1). Only 21 articles written in English language were included, where the titanium nanoparticles were incorporated into PMMA denture base material. The twenty-seven excluded articles did not fulfill the inclusion criteria. Few of the excluded articles were investigating the effect of surface coating with titania layer on physical properties (6 articles) and three others used microsized titania particles. Other excluded articles were investigating: a mixture of two nanofillers (ZrO_2_/TiO_2_), (Fe_2_O_3_/TiO_2_) (2 articles), the addition of barium titanate as a filler (3 articles), the addition of titania nanotubes (1 article), mica or zirconia/ABW (Aluminum borate whiskers) reinforced denture base acrylic (2 article), gallates modified titanium nanoparticles (1 article), and PMMA not used for denture bases (3 articles). Two excluded papers lacked full experimental data, three papers were reviews, and one paper was not in English language. The selected articles were reviewed to extract all data related to PMMA/TiO_2_ nanocomposite and to figure out the properties of TiO_2_NP, method of addition, interaction with PMMA resin matrix, effects on the properties of newly introduced nanocomposite, and finally evidence on their clinical performance.

### 1.1. Properties of *TiO*_2_NP

TiO_2_NP has proved to have antimicrobial properties. Moreover, it is a cheap biocompatible material, chemically stable, free of toxicity, resistant to corrosion with high strength, and high refractive index [8, 18, 23, 30]. Furthermore, the literature showed that even the slight addition of TiO_2_NP reinforcing agent to a polymeric material affects the electrical, optical, chemical, and physical properties of the resulting hybrid material [9, 31].

Its photocatalytic ability promoted it to be known as an antimicrobial agent encouraging its addition to biomaterials [11, 12, 20, 23, 25]. TiO_2_NP have been found effective against a wide range of microorganisms including gram-positive and gram-negative bacteria, fungi, and viruses [11, 23, 25]. The antimicrobial effect could be attributed to the surface properties and structure of the nanoparticles, including; nanocrystalline TiO_2_, hydrophilic surface effect, infrared reflectivity, and noncontact antimicrobial activity. Therefore, TiO_2_NP have been recommended as filler in polymeric materials [24].

### 1.2. Methods of PMMA/*TiO*_2_ Nanocomposite Preparation

Resulting properties of the hybrid material (PMMA/TiO_2_NP) will depend on the dispersion of the nanoparticles within the matrix, which is directly related to the added amount [9]. To achieve good dispersion of nanoparticles within polymers, different methods of TiO_2_NP addition were suggested. It is either added to acrylic powder or monomer. The addition of TiO_2_NP to acrylic powder was suggested where the required percentages were weighed and thoroughly mixed with acrylic powder. To attain a uniform mixture and homogenous distribution of TiO_2_NP, ultrasonic mixer, mortar, and pestle, high-energy ball milling and silanization of particles were all employed in which ball milling seemed to be the most effective method [16, 19, 21–23]. In another method, TiO_2_ nanoparticles were mixed with the acrylic powder up to 20 min in an amalgamator to obtain a homogenous mix [15, 21]. Others mixed the nanoparticles with resin powder by hand to create the desired filler/powder ratio [13, 14].

Addition of TiO_2_NP to liquid monomer is another method of filler incorporation within acrylic resin [22]. Investigators added nanoparticles to acrylic monomer to prepare monomer/TiO_2_NP in different concentrations. To insure uniformity, ultrasonic dispersion was done [10, 24]. The monomer containing NP was sonicated for 60 min before mixing with PMMA powder [32]. Others were sonicated using a probe at 120 W and 60 KHz for 3 min to prevent nanoparticles agglomeration and insure homogeneity of the mixture [18, 25].

An additional method called twin-screw extraction process was developed to disperse the particles into the PMMA [9, 33, 34]. TiO_2_NP was mixed with the acrylic and extruded using ZSK-25 twin-screw extruder at 210°C and screw speed of 250 rpm. Nanocomposite granules were then dried at 80°C for 2 h using dryer unit of injection molding machine [34].

Hence, the properties of nanocomposites depend on the interactions between the polymer matrix and the filler, suggesting the importance of functionalized TiO_2_NP [35, 36]. As revealed by a previous study, PMMA nanocomposite based on functionalized TiO_2_NP demonstrated better mechanical and physical properties [30]. One reported method of silanization used 100 ml of ethanol solution (70 vol%) with adjusted pH of 4.5 through titrating with 99.9% acetic acid. Following that, silane-coupling agent (TMSPM) was added to ethanol solution and mixed. A hundred grams of TiO_2_NP were added to TMSPM-ethanol solution and mixed for 20 minutes followed by probe sonication for 30 minutes. The mixture was then left to dry for 14 days and the resulting powder was a silanized TiO_2_NP [18, 37]. Others modified the titania nanoparticles using methacrylic acid (MA) by dispersing 10 g of nanoparticles into 250 ml iso-propanol and 125 ml of MA into 80°C ultrasonic bath for 8h. The resulting compound was dried at 85°C after infiltration and washing [14].

### 1.3. Behavior of *TiO*_2_NP toward PMMA

In addition to NP size and shape, its interaction with PMMA matrix is considered a main factor of reinforcement effectiveness, which depends on the surface characterization of the NP. Chatterjee (2010) studied the interaction between PMMA and TiO_2_NP and found that they interact chemically and physically [9]. The TiO_2_NP can react with -COOR group of PMMA polymer in two different ways. One way is the formation of H-bond between carbonyl group (-C=O) and surface hydroxyl group (-OH) of TiO_2_NP. The other way is the binding of TiO_2_NP with two oxygen atoms of -COOR by a bidentate coordination to Ti^4+^ cation [9]. When TiO_2_NP come to the surface, they form crosslinks with PMMA [9]. As the amount of TiO_2_NP loading within the PMMA increases, this bonding also increases. All this is possible because of the hydroxyl group on the surface of TiO_2_NP and the -C=O (Carbonyl), -OH (Hydroxyl), -COOH (Carboxyl), and -COOR (Ester) groups in the polymer matrix. The cross linking has been confirmed by Fourier-transform infrared spectroscopy (FTIR) [22, 33].

The dispersion of the TiO_2_NP within the matrix hinders polymer chain movements due to the strong adhesion between the TiO_2_NP and PMMA. As a result, better modulus is seen with TiO_2_NP-PMMA composite materials [9]. Other investigators reported increased stiffness of the composite material and decreased PMMA mobility [19]. This may be due to the large interface of TiO_2_NP when wrapped in PMMA, which possesses a smaller dielectric coefficient. There will be a strong electric interaction between them resulting in an electric dipole layer at the nanoparticle surface. In terms of the atomic bonding, the origin of the nonlinear refractive index may be due to the hyperpolarizability of Ti-O pairs [21, 38]. Moreover, the addition of spherical nanoscale particles is capable of filling the interpolymeric spaces, which was found to enhance the polishability of the material [15].

### 1.4. Properties of PMMA/*TiO*_2_ Nanocomposite

As mentioned earlier, PMMA is the most predominant polymer-based material for dentures. However, its mechanical and surface properties are low [39]. The addition of any material to improve the antimicrobial properties of the PMMA should not have an adverse effect on the mechanical properties. In reverse, it is highly preferred to add a material that will improve both biological and mechanical properties [34]. TiO_2_NP is commonly used as a coloring agent and filler. It is capable of increasing the toughness of polymers producing a composite material with superior properties [40]. With the added antimicrobial property, it is incorporation into PMMA resins is extensively used in oral removable appliances [41]. Therefore, the properties of the nanocomposite were screened in this review to evaluate the reliability of TiO_2_NP as an additive to improve the performance of PMMA/TiO_2_ nanocomposite denture base material.

#### 1.4.1. Antimicrobial Activities

TiO_2_NP were found to have an intrinsic antimicrobial property due the production of cytotoxic oxygen radicles [26]. TiO_2_NP are ideal additives because of their nontoxicity and chemical stability. However, under certain conditions they have an antimicrobial ability [41]. When they are exposed to UV-light in the presence of oxygen and water, they decompose and oxidize other compounds; organic and inorganic. Therefore, they can be considered as antimicrobial additive [41]. Furthermore, TiO_2_NP had the ability to inhibit the adherence of cariogenic bacteria in planktonic phase as well as in later phases of biofilm formation. The addition of TiO_2_NP to PMMA resin used for dentures or other oral appliances will have a positive effect against microbial colonization [42].

Anehosur et al. (2012) found that the addition of 3 wt% TiO_2_NP to PMMA produced a positive antimicrobial effect. It had the ability to reduce microbial number, which prevents quorum sensing thereby halts plaque formation on PMMA/TiO_2_ nanocomposite surface [11]. Due to the photocatalytic property against microorganisms, patients will be able to maintain the hygiene of their dentures easily by exposing them to solar energy/light sources to activate the TiO_2_NP [11]. On the same way, Alrahlah et al. (2018) reported a 50% and 90% decrease in bacterial cell attachment of* E. faecalis* and* P. aeruginosa,* respectively, with the mere addition of 3% TiO_2_NP [43]. Likewise, Totu et al. (2017) investigated the effect of incorporating TiO_2_NP into a 3D-printed PMMA denture in an attempt to improve denture antimicrobial characteristics and found that even the small addition of 0.4% of TiO_2_NP to PMMA resulted in a nanocomposite that prevented the colonization of microorganisms and further formation of biofilm [24]. As a result, TiO_2_NP could be incorporated into denture PMMA resin to successfully fabricate antimicrobial dentures [24]. The large active surface area of the nanoparticles compared to their small size makes them highly effective in low percentages of addition. It was found that as low as 0.4%, 1% and 2.5% TiO_2_NP inhibited the growth of* Candida*. As reported in the literature, TiO_2_NP caused a halt in the cellular enzymes and increased cell wall permeability causing cell death [44].

Few studies reported that the addition of as much as 5wt% TiO_2_NP to the PMMA is needed to achieve the antimicrobial effect [45]. However, with this addition, structural decomposition and material weakening may occur [26].

#### 1.4.2. Surface Properties

Surface microtopography of the denture is a very important feature in microbial adhesion and plaque formation and subsequently denture stomatitis.

*(1) Surface Roughness. *Denture wearers have high prevalence of poor oral health. This is mostly caused by the surface contours of the dentures and the irregularities of the oral mucosa [45]. Many factors were found to have an effect on the surface roughness (R_a_) of denture bases, one of which is the reinforcing material of the acrylic resin [46]. Alwan and Alameer (2015) found an increase in surface roughness with the addition of 3wt% of TiO_2_NP to PMMA denture base material and attributed this increase to the presence of nanoparticles on the surface of specimens [18].

To establish a relation between contact angle (wetting) and the amount of TiO_2_NP filler in the PMMA resin, Hashem et al. (2017) found that it is dependent on filler amount. The addition of 1wt% TiO_2_NP led to a reduction in surface wetting while the addition of more filler improved wetting. This finding confirms the effect of fillers on the surface of composite material compared to the pure counterpart [22].

While few investigations were made on surface roughness of PMMA/TiO_2_ nanocomposite, a significant increase was reported compared to pure PMMA [18]. As the surface gets rougher and R_a_ values increase, the surface area increases, meaning more sites are available for microbial adhesion and colonization. One of the most common organisms to be harbored on the surface of acrylic denture bases is* C. Albicans *[47]. Studies have reported that the surface roughness of a denture should not exceed 0.2 *μ*m [45]. Although there is a direct relation between surface roughness and* candida* adhesion (increased roughness increases* candida* adhesion), the reduction in* candida *count confirms the antifungal properties of TiO_2_NP.

*(2) Hardness. *Reports have confirmed that TiO_2_NP improved the hardness of heat cure PMMA [10, 18]. Xia et al. (2008) attributed the increase in surface hardness to two factors: proper filler content and use of silane-coupling agent which increases the bonding between filler and resin matrix [48]. The effect on surface hardness was detected with additions of TiO_2_NP as low as 1% [21]. In another study investigating the addition of 0.5%, 1%, and 2%, hardness values were found to increase with the addition and the highest values were seen with 2wt% TiO_2_. Mosalman et al. (2017) related this to increased number of bonds between matrix and fillers, which requires more energy to break these bonds [49]. Hashem et al. (2017) reported an increase in hardness values that reached 20%, 30%, and 34% more than pure PMMA with 1%, 2%, and 3% TiO_2_NP, respectively. This was explained by the increase in material stiffness due to presence of rigid particles within the matrix in addition to reduction of matrix mobility [22].

Alwan and Alameer (2015) concluded that 3% addition of TiO_2_ caused a significant increase in surface hardness compared to pure PMMA [18] while Ahmed et al. (2016) suggested the need of adding 5% TiO_2_NP to increase surface hardness of conventional and high impact heat cure acrylic resin to remarkable values [19]. More recently, Alrahlah et al. (2018) confirmed the increase in surface hardness of up to 35% with 3% TiO_2_NP addition. The values of the hardness measurements were higher at the surface of the composite resin and decreased as we move inward toward the core of the material, suggesting higher crosslinking at the surface [43]. Regarding selection of the most appropriate concentration of filler for inclusion within denture resin, results of multicriteria decision making (MCDM) method suggested that a filler content in the range of 2wt% would create a composite material with improved mechanical properties including surface hardness [34].

#### 1.4.3. Mechanical Properties

*(1) Flexural Properties. *During function, oral appliances including dentures are exposed to a magnitude of deforming stresses and any factor that increases the deformation of the denture base may lead to fracture [17]. One of the factors that may change the amount of denture deformation is the additive into the PMMA resin. The effect of TiO2NP addition on flexural strength of PMMA is partially dependent on the type of acrylics and the concentration of nanoparticles [19]. In* 2013*, Sodagar et al. investigated the effect of adding 0.5% and 1% TiO2NP into PMMA and reported a decrease in flexural strength. PMMA containing 0.5wt% showed the lowest values. It was concluded that there is an inverse relation between the concentration of the filler and the flexural strength of reinforced PMMA [12]. Han et al. (2008) reached the same conclusion and related the results to agglomeration of particles within the matrix, which makes them stress concentrations areas [50]. Other studies also reported a reduction in flexural strength [13, 19, 32] and modulus of elasticity [51] with the addition of TiO_2_NP. Hamouda et al. (2014) found that TiO_2_NP caused a reduction in flexural strength with no change in flexural modulus [13]. In the same token, Nazirkar et al. in (2014) were exploring the effect of adding TiO_2_NP into heat cure acrylic resin on antimicrobial properties and reported an adverse effect on flexural strength of the final product [32].

In contrast to previous studies, superior flexural properties were reported with different concentrations of TiO_2_NP added to PMMA than those of normal PMMA [10, 18, 21, 52]. This improvement may be attributed to the effect of silanization of TiO_2_NP [18] or the good dispersion of fillers within the matrix, which improves modulus, transverse strength, and ductility [18, 52]. Hashem et al. (2017) found that, with 0.5–3wt% TiO_2_NP addition, flexural strength and modulus increased. Moreover, they suggested that reinforced PMMA could offer superior chemical and mechanical properties making it a better option for dentures than pure PMMA resin [22]. On the same way, Rashahmadi et al. (2017) found that the mere addition of 0.5% TiO_2_NP improved the flexural strength and Young's modulus by 4%. Based on MCDM results, TiO_2_NP addition to PMMA is an excellent option for improving properties of PMMA for dental applications especially in 2wt% concentration [34]. In a recent study by Karci et al. (2018), [16] different types (heat- auto-, and microwave polymerized) of acrylic mixed with 1%, 3%, and 5% nanotio2 were used to test the flexural strength of the resulting composite. That study came to a conclusion that the addition of 1% TiO_2_NP to heat- and autopolymerized acrylic could improve the flexural strength while it remained unchanged with 3% TiO_2_NP and decreased with 5% for all types of acrylic. This was explained by the increase in agglomeration of nanoparticles at higher addition percentages.

In a study by Mosalman et al. (2017), various percentages of TiO_2_NP (0.5, 1, and 2wt %) were added to pure PMMA and found that the flexural strength of all groups stayed unchanged. Samples with 0.5wt% TiO_2_NP showed only 3.75% improvement in flexural strength compared to pure PMMA. Young's modulus for all groups was improved with the highest value seen in samples containing 2wt% TiO_2_NP [49].

*(2) Impact Strength. *With regard to impact strength, the addition of TiO_2_NP to heat cured acrylic resin resulted in a positive effect compared to pure PMMA [18]. Studies have confirmed this finding with different concentrations of TiO_2_NP, including 1% [19, 23], 2wt%[34, 49], and 3wt% [25]. The same finding was reported after the addition of silanized TiO_2_NP [53]. The detected increase in impact strength was justified by good bonding between PMMA matrix and TiO_2_ nanofillers. These fillers reside in small voids between polymer chains and result in slow segmental motion. Also, they provide large surface area due to their small size, which helps in energy dissipation [25]. Others suggested that the nanofillers in the acrylic resin tolerate most of the applied load while the resin matrix helps in structural integrity and load distribution, which eventually inhibits crack propagation [23].

*(3) Tensile Strength. *Shirkavand and Moslehifard (2014) investigated the effect of 0.5wt%, 1wt%, and 2wt% TiO_2_NP on tensile strength of PMMA base resins and found that the tensile strength of nanocomposite was the highest with 1wt% TiO_2_NP and the improvement was 35% more compared to pure PMMA. However, further increase in TiO_2_NP content led to an adverse effect [15] due to clustering of TiO_2_NP. The fillers work as impurities and act as defects and stress concentration centers [15, 49]. The same results were also reported by Ghaheremani et al. (2017) [23]. The increase in tensile strength is probably attributed to the fact that, following the incorporation of nanoparticles into acrylic powder, the applied load is mainly tolerated by these nanoparticles.

Contrary to previous studies, Chatterjee in 2010 found that nanocomposite with filler content as high as 5wt% and 15wt% had a 59% and 95% increase in tensile modulus, respectively, compared to that of pure PMMA [9]. This was caused by the strong adhesion between TiO_2_NP and PMMA. The applied load is transferred through these interfacial surfaces to the strongest material that is the nanoparticles [9].

#### 1.4.4. Color Stability

It is noteworthy that the reinforcing filler material should ideally improve the mechanical properties without causing an adverse reaction to the aesthetics [25, 41]. TiO_2_NP have whitish color; therefore, the appropriate percentage of this additive that fulfills the aforementioned requirement should be considered. An objective evaluation of the color change is usually done using a spectrophotometer where the difference between light absorption between different samples is calculated [28]. Aziz in 2018 evaluated the color change after adding 3wt% of TiO_2_NP and found that the amount of light absorbed increased making the reinforced specimens more opaque compared to pure PMMA. This change was due to the presence of TiO_2_NP within the matrix, which absorbs more light than polymer matrix due high atomic number [25]. Further studies are required to determine the proper TiO_2_NP percentage needed to attain a PMMA/TiO_2_ nanocomposite with superior properties and acceptable aesthetics.

#### 1.4.5. Water Sorption, Solubility, and Porosity

Conventional acrylic resins have voids and porosities that allow water molecules exchange which could be the leading cause for water sorption and solubility [18]. The nature of denture resin material allows it to absorb water, which acts as a plasticizer and affects the material dimensional stability and denture durability [41]. The water molecules force themselves between polymer chains, move them apart, create internal stresses, and result in crack formation [29]. As reported by Alwan and Alameer [18], the addition of TiO_2_NP to heat cure acrylic resin decreases water sorption and solubility significantly compared to pure PMMA. Torres et al. (2011) concluded that PMMA-TiO_2_-Fe_2_O_3_ nanocomposites had lower sorption and porosity values compared to pure PMMA but similar solubility levels. However, the solubility levels were originally low, which is important to prevent the adverse effects on oral structures or polymer functions [41]. Also, as the material gets more homogenous, it becomes less soluble with lower water sorption levels [29].

Therefore, the addition of TiO_2_NP fills the microvoids and polymer interstitial spaces decreasing the ability of composite material to absorb water. In addition to that TiO_2_NP are insoluble in water and these particles partly replace the hydrophilic matrix, which decreases water uptake. The use of silane-coupling agent in silanization process of TiO_2_NP could lead to a reduction in the amount of water that reaches the inner layers of polymer matrix [18].

#### 1.4.6. Thermal Properties

The addition of 3% TiO_2_NP to PMMA had no effect on thermal conductivity [25]. The glass transition temperature (T_g_) test is done to understand the thermal stability and determine the temperature at which the material starts to degrade. As the percentage of TiO_2_NP increases, the T_g_ of nanocomposite increases linearly up to 7.5wt% TiO_2_. Pure PMMA is stable up to 134°C (1% weight loss) while PMMA/2wt%TiO_2_ and PMMA/5wt%TiO_2_ are stable up to 154°C and 180°C, respectively. T_g_ was improved by 15%–34% with 2%–5% addition of TiO_2_NP, respectively. However, further addition of TiO_2_NP caused a decrease in thermal stability [33]. Another study by Safi (2014) reported the positive effect of TiO_2_NP addition on the T_g_ temperature of acrylic resin [51]. In a recent study [43], authors evaluated the effect of adding 1%, 2%, and 3% TiO_2_NP to PMMA on thermal behavior and found a slight effect on T_g_, degradation temperature, and rate.

Chatterjee (2010) in another study reported that the addition of 5wt% TiO_2_NP caused a 23% increase in T_g_ and this improvement reached only 3% when the amount of TiO_2_NP was increased to 15% [9]. Also, pure PMMA showed 5% weight loss at 217°C while a higher temperature (310°C) was needed to create the same effect in the PMMA/5wt%TiO_2_ [9]. Recently, Totu et al. (2018) [14] confirmed the increase in decomposition temperature for PMMA/TiO_2_NP composite suggesting an improved thermal stability. This may be explained by the reduced intercrystalline distance, interaction, and bond formation between nanoparticles and polymer chains or the absorption of some energy by the titania particles increasing the thermal stability. Final decomposition of the PMMA/TiO_2_NP composite happened at temperatures 620-700°C with decreasing pattern of the total mass loss as the amount of nanoparticles increased.

The thermal stability of TiO_2_NP added to PMMA inhibits the degradation of the resin and improves the thermal stability of the composite material [33]. Another explanation for thermal stability is the migration of TiO_2_NP with low surface energy to the surface of the PMMA resin to form a heat resistant layer [9]. The decrease in thermal stability with higher amount of nanofiller may be attributed to the agglomeration of filler particles rather than forming a filler-to-matrix interaction. This agglomeration reduces the effect of heat retardation associated with TiO_2_NP [33]. TiO_2_NP crystalline phase has free electrons that can be associated with surface reactions. Oxygen that diffused in the nanocomposite samples was absorbed on the surface of TiO_2_NP. Hence, diffused oxygen amount in the PMMA matrix was lower than in pure PMMA, which led to the slower thermooxidative degradation of the PMMA matrix [9, 33].

It is knows that PMMA shrinks upon polymerization causing dimensional changes in the final product. Dimensional stability is also affected by coefficient of thermal expansion. The result of a study by Hashem et al. (2017)[22] showed that pure PMMA and PMMA/3%TiO_2_ started to disintegrate and lose weight at 200°C with 90% weight loss happening at 400°C for both materials, suggesting no significant effect of TiO_2_NP on the melting temperature of the composite material. This might be explained by the minimal amount of filler addition or the presence of large number of decomposition sites within the material [22].

#### 1.4.7. Viscoelastic Behavior

Little work has been done on the effects of TiO_2_NP on the viscoelastic behavior (creep-recovery and relaxation) behavior of PMMA matrix. Recently, in a study by Alrahlah done to investigate viscoelastic properties of TiO_2_NP-modified PMMA denture base composite, it was found that the creep-recovery and relaxation behaviors of PMMA were significantly improved due to the addition of TiO_2_NP. Also, the improvement further increased as the concentration of the nanofiller changed from 1% to 3%. This improvement in the behavior indicates the role of the nanoparticles in increasing the stiffness of the PMMA matrix owing to the reduction in its molecular mobility and free volume [43].

#### 1.4.8. Electrical Behavior

It is imperative to avoid prolonged contact between oral mucosa and materials with high electrical conductivity. Metallic particles present in restorative materials my produce a galvanic effect in the highly conductive oral environment causing oral discomfort, changes in cell proliferation, and immune markers [27, 54]. Totu and colleagues (2018) [55] studied the effect of adding different percentages of TiO_2_NP (0% (control), 0.2%, 0.4%, 0.6%, 1.0%, 2.0%, 2.5%, and 5%) to PMMA used for 3D printing on the electrical properties of the resulting nanocomposite. They reported a decrease in material resistance with the additions of ≥1% TiO_2_NP. Also, an increase in electrical conductivity was noted with the addition of 5% TiO_2_NP. However, the composite material still maintained its insulating property.

## 2. Overall Performance and Clinical Significance

It is well understood that advancements in biomaterial science affect the progression of technologies in any field including dental prostheses. The introduction of nanomaterials had significantly changed the clinical and technological aspects of dentistry. In this paper, the latest research progress on the applications of TiO_2_NP in prosthodontics was reviewed. It clearly shows varying responses of physical and mechanical properties of the modified materials where a number of properties improved, others deteriorated, and few did not change. Their level of effectiveness as shown in the literature is diverse, being more or less effective than pure materials. Therefore, to attain removable prostheses with improved properties and acceptable clinical performance, the material of manufacture (acrylic resin) can be enhanced by adding proper percentage of nanofiller, initial surface treatment of the nanoparticles, and appropriate selection of addition method. Authors hope that this review article would provide some valuable elicitation for future scientific and technological innovations in the related field.

Based on this review, TiO_2_NP were found to be enhancers in some aspects, modifiers in some, and insignificant in others. The effect depends mainly on NP size, addition method, surface treatment, and loading percentage. Although the size of NP ranged between 5 nm and 350 nm, the results of the studies were not justified based on the nanofiller size, and a clear link between size and effect was not established. For that, further investigations to relate the resulting properties to nanoparticle size are required.

Variations in the results were mainly related to filler mode of addition. The addition of nanoparticles to acrylic monomer was considered more effective owing to better dispersion of NP within the monomer. In this method, the dominant improvement was noticed in mechanical properties while physical properties were slightly affected. It is worth noting that with this technique the polymer: monomer ratio may be affected. Therefore, mixing the nanofiller with acrylic powder has been suggested and studied. Till now, no study can be found that compares between the effects of different modes of addition (nanofillers addition to powder or monomer). Based on this review, further investigations of the above-mentioned point are necessary as well as the proper way to establish the proportion of polymer/monomer ratio for each method; hence nanofiller addition interferes with the manufacture's recommended ratios.

The percentage of addition also plays a role in resulting properties. While the range of addition was very broad (0.5–30wt%), low percentages resulted in improved properties compared to higher percentages. Simple addition of 1–2wt% ratios exhibited improved properties, while increasing the filler content more than 5wt% significantly weakened the final nanocomposites. In fact, the bonding between TiO_2_NP and resin matrix is a critical factor to achieve the desired properties of nanocomposite. As showed in Table 1, the treatment of NP with saline coupling agents improved the properties in comparison to untreated particles. Therefore, NP surface treatment is recommended.

According to Table 1, inconsistencies in prepared specimen sizes for the same test were seen, which may have contributed to variations in the results between studies experimenting with the same filler percentage. This reflects a major error in the methodologies of the studies where the test should have been done according to standardized specimens' dimensions and testing procedures. Testing should follow an internationally accepted standardization protocol like the ADA specifications for denture base polymers to make it possible to compare results between different studies.

Titanium nanoparticles remain under focus because of the antimicrobial efficacy against* candida *species despite their negative effect on a number of properties. Incorporation of TiO_2_NP into PMMA matrix proved to have antimicrobial effects, specifically on* candida* species [24]. Not to be lost in this discussion is the effect of TiO_2_NP surface modification on antimicrobial properties of the final product. It was shown that modification with noble metals such as (Ag, Au, Pt, and Pd) on TiO_2_NP surface enhanced the photocatalytic ability of the nanoparticles and bactericidal activity thereafter [12].

Even with this number of studies on recently introduced TiO_2_NP into PMMA, no clear evidence on the clinical applicability of this nanocomposite has been demonstrated. Further investigations are required to interpret and confirm the chemical structure changes in PMMA/TiO_2_ nanocomposite and also to determine the need from otherwise for surface treatment as well as the proper percentage of addition that will not affect the final product adversely.

Research to improve upon existing nanomaterials is still ongoing with emphasis on efficiency. Although the science behind nanotechnology is intriguing, the lack of long-term evidence addressing their clinical performance restricts their wide clinical use. Overall, there is an essential requirement to investigate the durability of these PMMA/TiO_2_ composites in different environmental conditions to extend the applicability of these hybrid materials.

## 3. Conclusion

Based on the current review, we could conclude that the addition of TiO_2_NP to PMMA denture bases is still questionable for working as a reinforcing material and requires further investigations following the ADA specifications. These investigations must explore the chemical and structural changes happening in the nanocomposite after TiO_2_ addition. The improvements in properties mentioned above were dominantly seen with lower concentrations of TiO_2_ and the increase in the amount of added nanoparticles caused adverse effects on resulting PMMA/TiO_2_ composite.

In conclusion, there is an essential need to investigate the clinical performance and durability of these nanocomposites in different conditions simulating the oral environment to verify their applicability and provide an insight of possible future researches in this field.

## Figures and Tables

**Table 1:** TiO_2_NP applications in denture base and its effect on the tested properties.

Authors /year	Particle size	Addition percentage	Type of Acrylic	Nanocomposite preparation	Properties tested	Specimen size	Effects (Increase/Decrease/Unchanged)
Chatterjee, 2010 [9]	5 nm	0%-15wt%	PMMA from Scientific Polymer Products (Ontario, NY)	(i) Measured TiO_2_NP mixed with PMMA for 5-10 min (ii) DACA twin-screw extraction process at 190°C and 100 rpm. (iii) Acrylic mixed for 6-7 min and extruded 5 times	(i) Tensile modulus (ii) Dimensional stability (iii) Glass transition temperature (T_g_) (iv) UV absorption	10 x 6 x 0.3 mm 5 mg	(i) Improvement in tensile modulus. (ii) Increased thermal stability (iii) Increased T_g_ (iv) Improvement in UV absorption ⟶maximum at 2% TiO_2_NP

Chatterjee, 2010 [10]	5 nm	0% 2.0% 5.0% 7.5% 10.0% 15.0% 30.0%	PMMA from Scientific Polymer Products (Ontario, NY)	(i) Measured TiO_2_NP mixed with PMMA for 5-10 min (ii) DACA twin-screw extraction process at 190°C and 100 rpm. (iii) Acrylic mixed for 6-7 min and extruded 5 times	(i) Glass transition temperature (T_g_) (ii) Thermal stability (iii) Decomposition temperature	10 x 6 x 0.3 mm	(i) T_g_ increased linearly up to 7.5% TiO_2_NP. (ii) Thermal stability increased (2%-15% TiO_2_NP) (iii) Decomposition temperatures increased with filler content up to 10% TiO_2_NP.

Anehosur et al., 2012 [11]	31 nm “Anatase phase”	3.0% In addition to surface coating.	DPI heat cure acrylic resin, (India)	(i) Visible light activated TiO_2_NP were mixed with methyl methacrylate monomer.	(i) Microbial inhibitory effect against S. Aureus	5 x 5 x 2 mm	(i) 3w% of TiO2 shows antimicrobial activity against S. Aureus.

Sodagar et al., 2013 [12]	21 nm “Anatase phase”	0% 0.5% 1.0%	Selecta Plus (self-cure acrylic resin)	(i) TiO_2_NP were added to acrylic monomer.	(i) Flexural strength	50 x 10 x 3.3 mm	(i) Flexural strength decreased as the filler content increased.

					(i) Flexural modulus		(i) No change in flexural modulus.
Hamouda and Beyari, 2014 [13]	21 nm	5.0%	Conventional heat cure acrylic resin (Acroston, WHN, England) and high impact (Metrocryl Hi, Metrodent, LTD, England)	(i) TiO_2_NP were mixed thoroughly with acrylic powder by hand.	(ii) Flexural strength (iii) Toughness	65 x 10 x 2.5 mm	(ii) Flexural strength and toughness decreased.
					(iv) Monomer release		(iii) No difference between control and TiO_2_ reinforced regarding monomer release.

Nazirkar et al., 2014 [14]	7 nm “Anatase phase”	0% 0.5% 1.0%	DPI heat cure acrylic resin	(i) TiO_2_NP added to acrylic monomer.	(i) Flexural strength	65 x 10 x 3.3 mm	(i) Flexural strength decreased as the TiO_2_ amount increased.

Shirkavand and Moslehifard, 2014 [15]	<25 nm (average ~ 20.4 nm) “Anatase and Rutile phases”	0% 0.5% 1.0% 2.0%	Heat cure acrylic resin from Ivoclar Vivadent	(i) TiO_2_NP were mixed with the acrylic resin polymer in an amalgamator for 20 min.	(i) Tensile strength	60 x 12 x 4 mm	(i) Tensile strength and elastic modulus improved with 1% TiO_2_NP. (ii) 0.5% and 2% TiO_2_ were not significantly different from each other or control.

Harini et al., 2014 [16]		0% 1.0% 2.0% 5.0%.	Clear heat cure acrylic resin	(i) Nanoparticles were incorporated into monomer by ultrasonic dispersion.	(i) Flexural strength	65 x 10 x 3 mm	(i) Flexural strength improved with TiO_2_ addition, significant difference noticed with 5%.

Safi, 2014 [17]		5.0%	Heat cure denture base acrylic (Superacryl plus, Czechoslovakia)	(i) Nanoparticles added to monomer and sonically dispersed.	(i) Coefficient of thermal expansion (ii) Modulus (iii) Glass transition	15 x 6 mm Cylinders 65 x 10 x 2.5 mm Powder form (10g)	(i) Decrease in coefficient of thermal expansion. (ii) Decreased in modulus of elasticity (iii) Increased T_g_

Alwan, and Alameer, 2015 [18]	<50 nm size	0% 3.0% (i) Silanized with TMSPM	Heat cure acrylic resin	(i) Silanized TiO_2_NP were added to monomer and sonicated.	(i) Impact strength (ii) Transverse strength (iii) Hardness (iv) Surface roughness (v) Water sorption and solubility	80 x 10 x 4 mm 65 x10 x 2.5 mm 50 x 0.5 mm disc	(i) Increased (ii) Increased (iii) Increased (iv) Increased (v) Decreased

Ahmed et al., 2016 [19]	46 nm	0% 1.0% 5.0%	Conventional heat cure acrylic resin (Implacryl, Vertex) and high impact heat cure acrylic resin (Vertex-Dental, Netherlands)	(i) TiO_2_NP were added into acrylic resin.	(i) Flexural strength (ii) Impact strength (iii) Hardness	50 x 10 x 3 mm 60 x 6 x 4 mm 25 x 10 x 3 mm	(i) Decreased with TiO_2_ addition. (ii) Increased only for conventional acrylic resin modified by 1%. (iii) Increased with 5% addition TiO_2_NP for both types of acrylic.

Sodagar et al., 2016 [20]	21 nm	0.5% 1.0%	Selecta Plus (self-cure acrylic resin)	(i) Nanoparticles were added to acrylic monomer and stirred	(i) Antimicrobial properties	20 x 20 x 1 mm	(i) TiO_2_ reduced microbial growth at both concentrations at 90 min under UVA exposure (ii) Antimicrobial activity of TiO_2_ is time dependent

Ahmed et al., 2017 [21]	<25 nm	0% 0.5% 1.0%	Heat cure acrylic resin from Dentsply International Inc., (Chicago, IL, USA)	(i) TiO_2_NP were added to acrylic polymer and mixed using amalgam capsule.	(i) Flexural strength (ii) Fracture toughness (iii) Hardness	65 x 10 x 2.5 mm 65 x 10 x 2.5 mm 30 x 10 x 2.5 mm	(i) Increased with both filler percentages. (ii) No effect on fracture toughness of both filler percentages. (iii) Increased with 1% filler.

Hashem et al., 2017 [22]	90 nm	0% 1.0% 2.0% 3.0%	Self-cure acrylic resin from Eco-crylcold, Protechno, (Spain)	(i) TiO_2_NP were mixed with the monomer.	(i) Flexural modulus and flexural strength (ii) Hardness (iii) Surface wetting	30 x 8 x 1 mm 50 x 1 mm discs	(i) Increased linearly (ii) Increased. (iii) Reduced with 1% filler content and increased with higher percentages. (iv) T_g_ decreased with TiO_2_ addition

Ghahremani et al., 2017 [23]	20-30 nm “Anatase phase”	0% 1.0%	SR Triplex Hot, heat cure acrylic resin (Ivoclar Vivadent Inc. Schaan, Liechtenstein)	(i) TiO_2_NP were mixed with acrylic resin powder in an ultrasonic mixer.	(i) Tensile strength (ii) Impact strength	60 x 12 x 3.9 mm 75 x 10 x 10 mm	(i) Increased (ii) Increased

Totu et al., 2017 [24]	65-170 nm	0% 0.2% 0.4% 1% 2.5%	PMMA+PEMA for 3D printing (eDent 100, EnvisionTec GmbH Gladbeck, Germany)	(i) Nanoparticles were added into PMMA solution with continuous stirring and ultrasonic mixing for 1 hour.	(i) Antimicrobial effect (*Candida scotti*) (ii) Complete denture manufacturing using stereolithography		(i) 0.4, 1% and 2.5% inhibited candida growth (ii) PMMA/0.4%TiO_2_ composite successfully used for denture fabrication

Aziz, 2018 [25]	30 nm	0% 3.0%	High impact heat cure acrylic resin (Vertex-Dental, Netherlands)-	(i) TiO_2_NP were dispersed in monomer and sonicated at 120W and 60 KHz for 3 minutes.	(i) Impact strength (ii) Color stability (iii) Thermal conductivity	80 x 10 x 4 mm 35 x 15 x 0.5 mm 40 x 2.5 mm	(i) Increased (ii) Increased color stability for test groups (iii) No effect

Alrahlah et al., 2018 [26]	80-100 nm	0% 1% 2% 3%	Heat cure acrylic resin (Lucitone 550, Dentsply Int. Inc. Pa, USA)		(i) Hardness and modulus (ii) T_g_, degradation temperature and rate (iii) Creep-recovery and relaxation behavior (iv) Antibacterial adhesion	50 x 10 mm discs cut in different sizes for different tests 7 mg 5 x 10 mm	(i) Increased (ii) Slight increase with 2% nano-filler content (iii) Improvement in behavior (iv) Decrease in bacterial attachment content

Karci et al., 2018 [27]	13 nm	0% 1% 3% 5%	(i) Auto-polyerized (Heraeus Kulzer, Newbury Berkshire, UK) (ii) Heat-polymerized Heraeus Kulzer, Newbury Berkshire, UK. (iii) Microwave-polymerized (GC Dental, Tokyo, Japan)	(i) TiO_2_NP were mixed with acrylic resin powder using ball milling at 400 rpm for 2 hours	(i) Flexural strength	65 x 10 x 3 mm	(i) Increased for heat- and auto-polymerized acrylic at 1% (ii) Decreased for all types of acrylic at 5%

Totu et al., Totu et al., 2017 [28]	“Anatase phase”	0% 0.2% 0.4% 0.6% 1.0% 2.5%	(i) PMMA-MA (ii) PMMA-MMA-BPO (iii) 3D printed PMMA (eD, EnvisionTec GmbH Gladbeck, Germany)	(i) TiO_2_ modified by methacrylic acid then manually mixed with PMMA mixture	(i) Thermal stability (ii) T_g_	Stereolithographic dentures	(i) Increased (improved) (ii) Increased

Totu et al., 2018 [29]	“Anatase phase”	0% 0.2% 0.4% 0.6% 1.0% 2.0% 2.5% 5.0%	PMMA for 3D printing (eDent 100, EnvisionTec GmbH Gladbeck, Germany)		(i) Resistance (ii) Electrical conductivity (iii) Dielectric constant		(i) Decreased with 1.0%, 2.0%, 2.5% and 5% (ii) Increased but material still maintained insulating properties (iii) Increased with 5%

## References

[1] Sakaguchi R. L., Powers J. M. (pp. 163-176, 2012). *Craig's Restorative Dental Materials*.

[2] Murakami N., Wakabayashi N., Matsushima R., Kishida A., Igarashi Y. (2013). Effect of high-pressure polymerization on mechanical properties of PMMA denture base resin. *Journal of the Mechanical Behavior of Biomedical Materials*.

[3] Adhikari R., Michler G. H. (2009). Polymer nanocomposites characterization by microscopy. *Polymer Reviews*.

[4] Navidfar A., Azdast T., Karimzad Ghavidel A. (2016). Influence of processing condition and carbon nanotube on mechanical properties of injection molded multi-walled carbon nanotube/poly(methyl methacrylate) nanocomposites. *Journal of Applied Polymer Science*.

[5] Chaijareenont P., Takahashi H., Nishiyama N., Arksornnukit M. (2012). Effect of different amounts of 3-methacryloxypropyltrimethoxysilane on the flexural properties and wear resistance of alumina reinforced PMMA. *Dental Materials*.

[6] Jordan J., Jacob K. I., Tannenbaum R., Sharaf M. A., Jasiuk I. (2005). Experimental trends in polymer nanocomposites—a review. *Materials Science and Engineering: A*.

[7] Gad M. M., Fouda S. M., Al-Harbi F. A., Näpänkangas R., Raustia A. (2017). PMMA denture base material enhancement: A review of fiber, filler, and nanofiller addition. *International Journal of Nanomedicine*.

[8] Li F., Zhou S., You B., Wu L. (2006). Kinetic study on the UV-induced photopolymerization of epoxy acrylate/TiO2 nanocomposites by FTIR spectroscopy. *Journal of Applied Polymer Science*.

[9] Chatterjee A. (2010). Properties improvement of PMMA using nano TiO2. *Journal of Applied Polymer Science*.

[10] Harini P., Mohamed K., Padmanabhan T. V. (2014). Effect of Titanium dioxide nanoparticles on the flexural strength of polymethylmethacrylate: An in vitro study. *Indian Journal of Dental Research*.

[11] Venkatesh Anehosur G., Kulkarni R. D., Naik M. G. (2012). Synthesis and Determination of Antimicrobial Activity of Visible Light Activated TiO2 Nanoparticles with Polymethyl Methacrylate Denture Base Resin Against Staphylococcus Aureus. *Journal of Gerontology & Geriatric Research*.

[12] Sodagar A., Bahador A., Khalil S., Saffar Shahroudi A., Zaman Kassaee M. (2013). The effect of TiO_2_ and SiO_2_ nanoparticles on flexural strength of poly (methyl methacrylate) acrylic resins. *Journal of Prosthodontic Research*.

[13] Hamouda I. M., Beyari M. M. (2014). Addition of glass fibers and titanium dioxide nanoparticles to the acrylic resin denture base material: comparative study with the conventional and high impact types. *Oral Health and Dental Management*.

[14] Totu E. E., Cristache C. M., Voicila E. (2017). On physical and chemical characteristics of Poly(methylmethacrylate) nanocomposites for dental applications. I.. *Materiale Plastice*.

[15] Shirkavand S., Moslehifard E. (2014). Effect of TiO2 nanoparticles on tensile strength of dental acrylic resins. *Journal of Dental Research, Dental Clinics, Dental Prospects*.

[16] Karci M., Demir N., Yazman S. (2018). Evaluation of Flexural Strength of Different Denture Base Materials Reinforced with Different Nanoparticles. *Journal of Prosthodontics*.

[17] Gurbuz O., Unalan F., Dikbas I. (2010). Comparison of the transverse strength of six acrylic denture resins. *Ohdmbsc Magazines*.

[18] Alwan S. A., Alameer S. S. (2015). The Effect of the Addition of Silanized Nano Titania Fillers on Some Physical and Mechanical Properties of Heat Cured Acrylic Denture Base Materials. *Journal of Baghdad College of Dentistry*.

[19] Ashour Ahmed M., El-Shennawy M., M. Althomali Y., Omar A. A. (2016). Effect of Titanium Dioxide Nano Particles Incorporation on Mechanical and Physical Properties on Two Different Types of Acrylic Resin Denture Base. *World Journal of Nanoscience and Engineering*.

[20] Sodagar A., Khalil S., Kassaee M. Z., Shahroudi A. S., Pourakbari B., Bahador A. (2016). Antimicrobial properties of poly (methyl methacrylate) acrylic resins incorporated with silicon dioxide and titanium dioxide nanoparticles on cariogenic bacteria. *Journal of Orthodontic Science*.

[21] Ahmed M. A., Omar A. A., El-Shennawy M., Ebrahim M. I., Althomali Y. M. (2017). Influence of addition of different types of nano-fillers on the microstructure and mechanical properties of PMMA based denture resin. *Kasmera Journal*.

[22] Hashem M., Al Rez M. F., Fouad H. (2017). Influence of titanium oxide nanoparticles on the physical and thermomechanical behavior of poly methyl methacrylate (pmma): A denture base resin. *Science of Advanced Materials*.

[23] Ghahremani L., Shirkavand S., Akbari F., Sabzikari N. (2017). Tensile strength and impact strength of color modified acrylic resin reinforced with titanium dioxide nanoparticles. *Journal of Clinical and Experimental Dentistry*.

[24] Totu E. E., Nechifor A. C., Nechifor G., Aboul-Enein H. Y., Cristache C. M. (2017). Poly(methyl methacrylate) with TiO2 nanoparticles inclusion for stereolitographic complete denture manufacturing − the fututre in dental care for elderly edentulous patients. *Journal of Dentistry*.

[25] Aziz H. K. (2018). TiO-Nanofillers Effects on Some Properties of Highly- Impact Resin Using Different Processing Techniques. *The Open Dentistry Journal*.

[26] Tsuji M., Ueda T., Sawaki K., Kawaguchi M., Sakurai K. (2016). Biocompatibility of a titanium dioxide-coating method for denture base acrylic resin. *Gerodontology*.

[27] Podzimek S., Tomka M., Sommerova P., Lyuya-Mi Y., Bartova J., Prochazkova J. (2013). Immune markers in oral discomfort patients before and after elimination of oral galvanism. *Neuroendocrinology Letters*.

[28] Kiat-amnuay S., Beerbower M., Powers J. M., Paravina R. D. (2009). Influence of pigments and opacifiers on color stability of silicone maxillofacial elastomer. *Journal of Dentistry*.

[29] Tuna S. H., Keyf F., Gumus H. O., Uzun C. (2008). The evaluation of water sorption/solubility of various acrylic resins. *European Journal of Dental Education*.

[30] Khaled S. M., Sui R., Charpentier P. A., Rizkalla A. S. (2007). Synthesis of TiO_2_-PMMA nanocomposite: using methacrylic acid as a coupling agent. *Langmuir*.

[31] Reijnders L. (2009). The release of TiO2 and SiO2 nanoparticles from nanocomposites. *Polymer Degradation and Stability*.

[32] Nazirkar G., Bhanushali S., Singh S., Pattanaik B., Raj N. (2014). Effect of Anatase Titanium Dioxide Nanoparticles on the Flexural Strength of Heat Cured Poly Methyl Methacrylate Resins: An In-Vitro Study. *Journal of Indian Prosthodontist Society*.

[33] Chatterjee A. (2010). Effect of nanoTiO_2_ addition on poly(methyl methacrylate): an exciting nanocomposite. *Journal of Applied Polymer Science*.

[34] Rashahmadi S., Hasanzadeh R., Mosalman S. (2017). Improving the Mechanical Properties of Poly Methyl Methacrylate Nanocomposites for Dentistry Applications Reinforced with Different Nanoparticles. *Polymer—Plastics Technology and Engineering*.

[35] Yuwono A. H., Liu B., Xue J. (2004). Controlling the crystallinity and nonlinear optical properties of transparent TiO_2_-PMMA nanohybrids. *Journal of Materials Chemistry*.

[36] Pandey J. K., Raghunatha Reddy K., Kumar A. P., Singh R. P. (2005). An overview on the degradability of polymer nanocomposites. *Polymer Degradation and Stability*.

[37] Tao C., Jianping S., Weng J., Ting L., Xiaozhu H. (2007). Preparation and properties of PMMA/TiO2 nanocomposite. *Chemical Reaction Engineering and Technology*.

[38] Yuwono A. H., Xue J., Wang J. (2003). Transparent nanohybrids of nanocrystalline TiO2 in PMMA with unique nonlinear optical behavior. *Journal of Materials Chemistry*.

[39] Alla R., Raghavendra K. N., Vyas R., Konakanchi A. (2015). Conventional and contemporary polymers for the fabrication of denture prosthesis: Part I overview, composition and properties. *International Journal of Applied Dental Sciences*.

[40] Trapalis C. C., Keivanidis P., Kordas G. (2003). TiO2(Fe^3+^) nanostructured thin films with antibacterial properties. *Thin Solid Films*.

[41] Acosta-Torres L. S., López-Marín L. M., Núñez-Anita R. E., Hernández-Padrón G., Castaño V. M. (2011). Biocompatible Metal-Oxide Nanoparticles: Nanotechnology Improvement of Conventional Prosthetic Acrylic Resins. *Journal of Nanomaterials*.

[42] Bahador A., Khalil S., Pourakbari B. (2014). Photocatalytic Effects of Acrylic Resins Incorporated with Nano-titanium Dioxide on Planktonic and Biofilm Growth of Four Cariogenic Bacteria. *Annual Research & Review in Biology*.

[43] Alrahlah A., Fouad H., Hashem M., Niazy A., AlBadah A. (2018). Titanium Oxide (TiO2)/Polymethylmethacrylate (PMMA) Denture Base Nanocomposites: Mechanical, Viscoelastic and Antibacterial Behavior. *Materials*.

[44] Kubacka A., Ferrer M., Cerrada M. L. (2009). Boosting TiO2-anatase antimicrobial activity: Polymer-oxide thin films. *Applied Catalysis B: Environmental*.

[45] Cheng Y., Sakai T., Moroi R. (2008). Self-cleaning ability of a photocatalyst-containing denture base material. *Dental Materials*.

[46] Bollen C. M., Lambrechts P., Quirynen M. (1997). Comparison of surface roughness of oral hard materials to the threshold surface roughness for bacterial plaque retention: a review of the literature. *Dental Materials*.

[47] Elshereksi N., Ghazali M., Muchtar A., Azhari C. (2017). Effect of nanobarium titanate addition on the surface characteristics of denture base resin. *International Journal of Mechanical and Production Engineering*.

[48] Xia Y., Feimin Z., Xie H., Gu N. (2008). Nanoparticles reinforced resin based dental composites. *Journal of Dentistry*.

[49] Mosalman S., Rashahmadi S., Hasanzadeh R. (2017). The effect of TiO2 nanoparticles on mechanical properties of poly methyl methacrylate nanocomposites. *International Journal of Engineering, Transactions B: Applications*.

[50] Han Y., Kiat-amnuay S., Powers J. M., Zhao Y. (2008). Effect of nano-oxide concentration on the mechanical properties of a maxillofacial silicone elastomer. *The Journal of Prosthetic Dentistry*.

[51] Safi I. N. (2014). Evaluation the effect of nano—fillers (TiO_2_ , AL_2_O_3_ , SiO_2_) addition on glass transition temperature, E-moudulus and coefficient of thermal expansion of acrylic denture base material. *Journal of Baghdad College of Dentistry*.

[52] Wang W., Liao S., Zhu Y., Liu M., Zhao Q., Fu Y. (2015). Recent Applications of Nanomaterials in Prosthodontics. *Journal of Nanomaterials*.

[53] Sun L., Gibson R. F., Gordaninejad F., Suhr J. (2009). Energy absorption capability of nanocomposites: A review. *Composites Science and Technology*.

[54] Podzimek Š., Hána K., Mikšovský M. (2008). The Influence of galvanic currents and voltage on the proliferation activity of lymphocytes and expression of cell surface molecules. *Folia Biologica*.

[55] Totu E. E., Voicila E., Pistritu V., Nechifor G., Cristache C. M. (2018). Evaluation of electrical characteristics for PMMA-TiO2 nanocomposites used in dentistry. *Evista de Chimie*.

